# Identification of the Putative Tumor Suppressor Characteristics of FAM107A *via* Pan-Cancer Analysis

**DOI:** 10.3389/fonc.2022.861281

**Published:** 2022-05-20

**Authors:** Dehua Ou, Zhiqin Zhang, Zesong Wu, Peilin Shen, Yichuan Huang, Sile She, Sifan She, Ming-en Lin

**Affiliations:** ^1^Department of Urology, The First Affiliated Hospital of Shantou University Medical College, Shantou, China; ^2^Clinical Medicine Science, Shantou University Medical College, Shantou, China; ^3^Department of Gynecology & Obstertrics, Zhongshan Hospital Xiamen University, Xiamen, China; ^4^Clinical Medicine Science, Guangdong Medical University, Zhanjiang, China

**Keywords:** FAM107A, pan-cancer, prognosis, methylation, immune infiltration, bladder cancer, renal cancer

## Abstract

Family with sequence similarity 107, member A(FAM107A) was supposed as a tumor suppressor for various types of tumors. However, no pan-cancer analysis of FAM107A is available. Therefore, we conducted a FAM107A-related pan-cancer analysis across thirty-three tumors based on TCGA database to explore the molecular characteristics of FAM107A. The FAM107A expression is reduced in most cancers, and its down-regulated expression was linked to poor overall survival and progression-free survival of tumor patients. Analysis of DNA methylation of the FAM107A gene showed a negative correlation between FAM107A expression and promoter methylation in numerous cancers. Furthermore, FAM107A expression was noted to be involved in myeloid-derived suppressor cell infiltration in multiple cancers. To explore the mechanism of FAM107A in cancers, KEGG, and GO enrichment analysis was performed and the result showed “cell adhesion” and “cAMP signaling pathway” terms as the potential impact of FAM107A on cancers. An experiment *in vitro* showed FAM107A knockdown promoted the proliferation, migration, and invasion of bladder cancer and renal cancer cells. Our study indicates that FAM107A may be a putative tumor suppressor in bladder cancer and other tumors.

## Introduction

According to statistics released by the International Agency for Research on Cancer (IARC), there were an estimated 19.3 million new tumor cases and nearly 10 million tumor death worldwide in 2020 (1). At this time, tumor as a genetic disease has become a consensus. Since the global situation of tumor morbidity and mortality is serious, it is urgent to identify cancer-related biomarkers that can be useful for tumor prevention and treatment. Early on, numerous oncogenes were identified through functional analysis of tumor genome by the known positive selection systems, with some tumor suppressor genes identified through analysis of the loss of heterozygotes (2–5). However, the understanding of the molecular mechanism of tumor occurrence and progression is still limited.

With the deepening exploration of cancer genomes, tumor molecular biological research has gradually come into the era of Pan-cancer analysis. Pan-cancer analysis refers to the simultaneous analysis of various types of tumor genomes to clarify common characteristics of tumors from different sources, which help to better understand tumors and provide broad-spectrum targets for clinical diagnosis and treatment of various tumors (6, 7). In the past decade, the Systematic Cancer Genome Project, including the Cancer Genome Atlas Project, has applied new sequencing technologies to analyze the whole genome of specific tumor types (8, 9). These specific techniques have identified new driving genes that lead to alterations in tumor biological properties, classified new molecular subtypes, and identified new biomarkers according to differences in genomics, transcriptomics, and proteomics. At present, massive clinical information and gene sequencing data of various tumor types, including transcriptome data, copy number variation, DNA methylation, and single nucleotide variation information, can be acquired for a variety of public databases, such as The Cancer Genome Atlas (TCGA), Gene Expression Omnibus(GEO), and the Oncomine database. The availability of these databases renders it expedient to perform a pan-cancer analysis.

Family with sequence similarity 107, member A (FAM107A), also known as Down-regulated in renal cell carcinoma 1 (DRR1) or Tohoku University cDNA clone A on chromosome 3 (TU3A), was firstly identified in the region of human chromosome 3p21.1, with a total length of about 10 KB and a transcript length of about 3.5 KB (10, 11). The encoded protein contains 144 amino acids, which acts as a stress-inducible actin-binding protein that participates in modulating actin filamentous (F-actin) dynamics (12). The results of gene homology analysis showed that FAM107A gene is highly homologous in human, mouse, rat, dog, cow, chicken, macaque, orangutan, and Xenopus laevis (13). The FAM107A gene is widely expressed in various normal tissues, especially in the nervous system. Sequencing analysis of amino acid showed that FAM107A has a coiled-coil (CC) domain and a nuclear location signal (10, 13). FAM107A was initially considered as a tumor suppressor. Its expression level decreased significantly or even disappeared in a variety of malignant tumors such as renal cell carcinoma, cervical cancer, colon cancer, laryngeal squamous cell carcinoma, prostate cancer, lung cancer, and Hodgkin’s lymphoma (10, 14–19). A number of studies have confirmed that FAM107A is involved in the process of tumorigenesis and tumor progression in some specific tumor types. Nevertheless, it is necessary to perform a pan-cancer analysis to clarify the molecular characteristics and potential role of FAM107A gene in cancers.

In this current study, a pan-cancer analysis of FAM107A gene expression characteristics and patient prognoses was conducted for the first time by using various public databases such as the TCGA project, Oncomine databases, GEPIA2, the Cbioportal, and the Clinical Proteomic Tumor Analysis Consortium (CPTAC). In addition, correlations between FAM107A expression, genetic mutation, DNA methylation, and immune infiltration were explored. Finally, the expression and potential biological functions of FAM107A were analyzed and verified *in vitro* experiments with bladder cancer cell lines.

## Materials and Methods

### Gene Expression Analysis of FAM107A

To observe the expression level and the expression difference of FAM107A between tumor and adjacent normal tissues for the different tumors or specific tumor subtypes, FAM107A was firstly investigated *via* the Oncomine dataset (https://www.oncomine.org/), a platform with the integrated results of high-quality standard tumor tissue microarray in the literature and the microarray database, as well as the analysis of 14 annotation databases. Besides, FAM107A was also input in the “Gene_DE” module of TIMER2 (Tumor immune estimation resource, version 2) website (http://timer.cistrome.org/) which is a website for analysis of gene expression of virous tumor types and tumor infiltrating immune cell components based on the data of the TCGA project (20). Next, the “Expression Analysis-Box Plots” module of the GEPIA2 (Gene expression profiling interactive analysis, version 2) website (http://gepia2.cancer-pku.cn/#analysis) (21), with the settings of P-value cutoff = 0.01, log_2_FC (Fold change) cutoff =1, and “Match TCGA normal and GTEx data”, was applied to obtain box plots of the expression difference between the tumor tissues and the corresponding normal tissues of the GTEx (Genotype-tissue expression) database for those certain tumors without normal tissue matched as controls in TIMER2 database. In addition, the “Pathological Stage Plot” module of GEPIA2 was selected to analyze the FAM107A expression in different pathological stages of all TCGA tumors *via* violin plots. In addition, we conducted FAM107A protein expression analysis of the CPTAC (Clinical proteomic tumor analysis consortium) dataset *via* the UALCAN portal (http://ualcan.path.uab.edu/analysis-prot.html), a comprehensive website for analysis of cancer OMICS data (22). Moreover, to further analyze the expression level of FAM107A in tumor cells, the Broad Institute Cancer Cell Line Encyclopedia (CCLE) website (https://portals.broadinstitute.org/ccle/about) was applied to visualize the FAM107A expression of diverse cancer cell lines.

### Survival Prognosis Analysis

According to the median expression level of FAM107A, tumor cases were divided into a high expression group and a low expression group to explore the relationship between FAM107A expression and the prognosis of different tumor patients. The “Survival Map” module of GEPIA2 (21) was used to obtain the overall survival (OS) and disease-free survival (DFS) significance map data of tumor patients with different FAM107A expression in TCGA database. By applying cutoff-high (50%) and cutoff-low (50%) values as the expression thresholds, all tumor cases were separated into the high-expression and low-expression cohorts. The Kaplan-Meier (K-M) survival plots were acquired by performing the log-rank test through the “Survival Analysis” module of GEPIA2 (21).

### Analysis of Genetic Alteration

The cBioPortal (https://www.cbioportal.org/) (23, 24), a website providing visualization, analysis, and download of large-scale cancer genomics data sets, was used for genetic alteration analysis of FAM107A. After logging into the web portal, the “TCGA Pan Cancer Atlas Studies” in the “Quick select” section was chosen. Then, “FAM107A” was input for queries of the genetic alteration characteristics of FAM107A. The “Cancer Types Summary” module was selected to scan the results of the alteration frequency, mutation type, and copy number alteration (CNA) in all TCGA tumors. After selecting the “Mutations” module, the mutational information of FAM107A protein can be displayed. The “Comparison” module was also selected to analyze the survival prognosis differences among the TCGA tumor cases with and without FAM107A genetic alteration. K-M plots of OS, DFS, progression-free survival (PFS), and disease-specific survival (DSS) were generated, respectively, with log-rank P-values.

### Analysis of DNA Methylation and Genomic Instability

The GSCALite (http://bioinfo.life.hust.edu.cn/web/GSCALite/) website (25) was applied to analyze the Spearman correlation between FAM107A mRNA expression and copy number variation (CNV), and generate the differential methylation bubble map of FAM107A between cancer tissues and adjacent tissues in 33 types of TCGA cancers. The diagram of correlation between expression and methylation was plotted. Moreover, DiseaseMeth version 2.0 (http://bioinfo.hrbmu.edu.cn/diseasemeth/) was applied to analyze the differential DNA methylation level of FAM107A gene in cancers and normal tissues. The correlation between expression of FAM107A and expression levels of DNA-methyltransferases, tumor mutational burden (TMB), microsatellite instability (MSI), and mutation levels of mismatch repair (MMR) genes was analyzed with SangerBox tool (http://sangerbox.com/).

### Analysis of Immune Infiltration

To explore the association between FAM107A gene expression and immune cell infiltration in TCGA datasets, the “Immune-Gene” module of the TIMER2 was used. To our knowledge, the TIDE (Tumor Immune Dysfunction and Exclusion) database was used to analyze the relationship between FAM107A expression and myeloid-derived suppressor cells (MDSCs). The partial correlation values and P-values were acquired through the purity-adjusted Spearman rank correlation test. Heatmap and scatter plot results were applied for result visualization. The correlation between expression of FAM107A and tumor purity-related factors (Stromal, Immune and ESTIMATE score) and key immune checkpoint genes across various cancers from the TCGA project was investigated with SangerBox tool.

### Analysis of FAM107A-Related Gene Enrichment

Firstly, the query of a single protein name (“FAM107A”) and organism (“Homo sapiens”) was used and searched in the STRING website (https://string-db.org/). Then, the reliable experimentally determined FAM107A-binding proteins were obtained after setting the following filters: minimum required interaction score [“Low confidence (0.150)”] and active interaction sources (“experiments”). Subsequently, the “Similar Gene Detection” module of GEPIA2 was used to obtain the top 300 FAM107A-correlated genes based on the datasets of all TCGA tumor samples. These genes were considered to have potential similar molecular functions as FAM107A and the “correlation analysis” module of GEPIA2 was applied to conduct a pairwise gene Pearson correlation analysis of FAM107A and FAM107A-correlated genes. The intersection of FAM107A-binding and correlated genes was visualized *via* a Venn diagram. Moreover, the two sets of genes were combined to perform gene ontology (GO) enrichment analysis and Kyoto Encyclopedia of Genes and Genomes (KEGG) pathway analysis by applying the “clusterProfiler” R package of R language software (https://www.r-project.org/), with two-tailed P <0.05 considered statistically significant. The results were finally visualized with the “tidyr” and “ggplot2” R packages.

### Cell Culture and Transfection

The immortalized normal human urothelial cell line SV-HUC1, the human bladder urothelial carcinoma (BLCA) cell lines UM-UC3, T24, 5637 cell lines, the human embryonic kidney cell line 293T, and the human renal cancer(RC) cell lines 786-O, 769-P cell lines were all purchased from the Chinese Academy of Sciences Committee on Culture Collection Cell Bank, Shanghai Institutes for Biological Sciences (Shanghai, China). SV-HUC1 cells were cultured in F-12K Nutrient Mixture (Gibco) supplemented with 10% fetal bovine serum(FBS) and 1% penicillin/treptomycin. UM-UC3 and 293T cells were cultured in DMEM (Gibco, USA) supplemented with 10% FBS and 1% penicillin/treptomycin. T24, 5637, 786-O and 769-P cells were cultured in RPMI 1640 medium(Hyclone) supplemented with 10% FBS and 1% penicillin/treptomycin. All the cells were cultured at 37°Cin a humidified incubator with 5% CO_2_. After purchasing the siRNAs against FAM107A and siRNA-control from GenePharma(RiboBio, Shanghai, China), the indicated cells were transfected with siRNAs assisted with the Lipofectamine 2000 according to the manufacturer’s instructions(Invitrogen, USA). The siRNA template sequences in our study were applied as following: siFAM107A#1, sense 5’-GCCAGAAUACAGAGAGUGGTT-3’, antisense 5’-CCACUCUCUGUAUUCUGGCTT-3’; siFAM107A#2, sense 5’-GCUGGAAUAGCAUCUCCUUTT-3’, antisense 5’-AAGGAGAUGCUAUUCCAGCTT-3’; siNC, sense 5’-UUCUCCGAACGUGUCACGUTT-3’, antisense 5’-ACGUGACACGUUCGGAGAATT-3’.

### RNA Extraction and qRT-PCR

Total RNA of cells was extracted with Trizol reagent(Invitrogen), and then cDNA was synthesized in accordance with manufacturer’s instruction(Accurate Biology, Hunan, China). Quantitative real-time PCR (qRT-PCR) was conducted to determine the mRNA expression using SYBR Green Permix Pro Taq HS qPCR kit (Accurate Biology) on the QS5 PCR machine (Applied Biosystems, USA). Gene expression levels were normalized against human housekeeping gene β-actin and calculated by the 2^-ΔΔCt^ relative quantification method. The specific primers in our study were applied as following: FAM107A, forward 5’- AGGGAGCGGGCAGACATTGG-3’ and reverse 5’-CACGGGGTTCAGCAGCTTCTTG-3’; GAPDH, forward 5’-GGTGAAGGTCGGAGTCAACG -3’, reverse 5’-CAAAGTTGTCATGGATGACC-3’.

### Cell Viability Assay

Cell proliferation was assessed by using Cell Counting Kit-8 reagent (Abcam, UK). Cells in the logarithmic growth phase were trypsinized and seeded at a density of 5×10^3^ cells/well in each well of a 96-well plate. After incubation at 37°Cfor different periods of time, CCK-8 reagent was added 10 μl into each well and the optical density values were measured at 460 nm using a microplate reader (Bio-Tek Instruments, Germany).

### Cell Colony-Formation Assay

We conducted cell colony-formation assay to evaluate the clonogenic ability of FAM107A-interfered BLCA and RC cells And 1,000 tumor cells were plated into 6-well plates and cultured for 10 days. For cell fixation, 4% paraformaldehyde was used and for staining, 2% crystal violet (Beyotime, China) was used. Finally, the cell colonies were counted and imaged.

### Wound Healing Assay

Cells were seeded in 6-well plates and cultured until the cells were basically 100% confluence. The cell monolayer was scraped by 200μl pipette and rinsed three times with PBS to form a clean wound. After incubation at 37°C for 24h, the closure of scratches was observed under an inverted microscope(Olympus). The corresponding photographs of scratch at 0h and 24h were taken under the inverted microscope. The cell-free area at 0h and 24h were measured by means of the Image J software (National Institutes of Health, Bethesda, MD, USA) and the percentage of wound closure was calculated.

### Transwell Assay

Transwell chambers (pore size, 8.0 μm; Biosciences, Heidelberg, Germany) coated with Matrigel (BD Biosciences, USA) at a concentration of 2 mg/ml and incubated at 4°C for 3h were used for cell invasion assay and 2×10^4^ cells were cultured on upper chamber with 100 μl serum-free medium. As chemo-attractant, 600 medium containing 20% FBS was supplemented into the lower chamber. After incubation for 48h, cells on the inferior surface of the transwell chamber were fixed with 4% paraformaldehyde and stained with 2% crystal violet. Photographs of five visual fields were taken under the inverted microscope (Olympus) and the number of invasive cells was counted.

### Western Blotting

The total protein was extracted from cells by using Radio Immunoprecipitation Assay (RIPA) buffer with the proteinase inhibitor. After centrifuged for 20min, the supernatants were collected and used for proteic concentration analysis with G250 solution. Twenty micrograms protein lysate were separated on 12% polyacrylamide gels and electrophoretically transferred onto polyvinylidene difluoride(PVDF) membrane. The membrane was blocked with Tris Buffered Saline with Tween(TBST) containing 5% skim milk for 1h at room temperature. Then, the membrane was blotted with the primary antibodies(anti-FAM107A, Absin, anti-β-actin, Cell Signaling Technology, USA) overnight at 4°C. On the second day, the membrane was washed with TBST three times and incubated with horseradish peroxidase (HRP) labeled IgG at room temperature for 1h. The immunoreactive bands were detected by using ECL Western blotting reagents with chemiluminescence detection system.

### Statistical Analyses

The data were displayed as means ± standard deviation(SD). SPSS ver. 23.0 software (SPSS, Inc., Chicago, IL, USA) was used for statistical analysis. Pairwise differences between the 2 groups was assessed by Student’s t test. The significance of differences among more than 2 groups was assessed using ANOVA analysis. P <0.05 was considered statistically significantly different.

## Results

### The Expression of FAM107A in Cancers and Cell Lines

**Figure 1:**
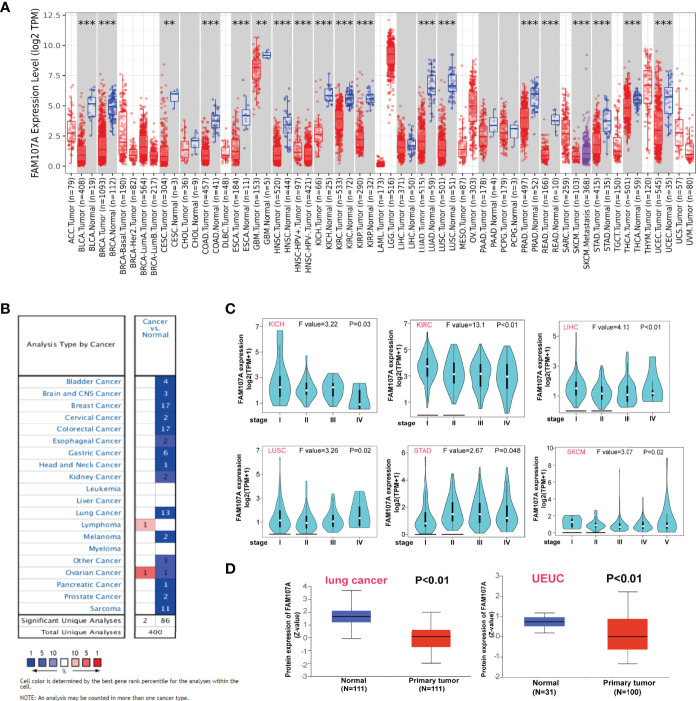
Expression level of FAM107A gene in pan-cancers and pathological stages. **(A)** The expression status of the FAM107A gene in different cancers or specific cancer subtypes was analyzed through TIMER2. **P < 0.01; ***P < 0.001. **(B)** The expression status of the FAM107A gene in different cancers was analyzed through Oncomine platform. **(C)** The expression levels of the FAM107A gene were analyzed by the main pathological stages of KICH, KIRC, LIHC, LUSC, STAD, and SKCM based on the TCGA data. **(D)** The expression level of FAM107A total protein in primary tumor tissue and normal tissue of lung cancer and UCEC was analyzed based on the CPTAC dataset.

To identify the differential expression of FAM107A gene between tumor and normal tissues, we firstly applied the TIMER2 approach to analyze the significance of FAM107A expression. The result (**Figure 1A**) shows lower expression(P<0.05) in BLCA (Bladder urothelial carcinoma), BRCA(Breast invasive carcinoma), CESC (Cervical squamous cell carcinoma and endocervical adenocarcinoma), COAD (Colon adenocarcinoma), ESCA(Esophageal carcinoma), GBM (Glioblastoma multiforme), HNSC(Head and Neck squamous cell carcinoma), KICH(Kidney Chromophobe), KIRC (Kidney renal clear cell carcinoma), KIRP(Kidney renal papillary cell carcinoma), LUAD (Lung adenocarcinoma), LUSC (Lung squamous cell carcinoma), PRAD(Prostate adenocarcinoma), READ (Rectum adenocarcinoma), STAD (Stomach adenocarcinoma), THCA (Thyroid carcinoma), and UCEC(Uterine Corpus Endometrial Carcinoma) than the corresponding normal tissues. Inversely, it was found highly expressed in lymphoma. The down-regulating expression of FAM107A between the normal tissues and tumor tissues was also detected in OV (Ovarian serous cystadenocarcinoma) and THYM(Thymoma) after including the normal tissues of the GTEx dataset as controls (**Figure S1**, P < 0.01). However, there was an up-regulating expression of FAM107A in TGCT (Testicular Germ Cell Tumors) and UCS (Uterine Carcinosarcoma) (**Figure S1**, P < 0.01). Besides, no significant difference of FAM107A expression between tumor and normal tissues was obtained significant for ACC (Adrenocortical carcinoma), DLBC (Lymphoid Neoplasm Diffuse Large B-cell Lymphoma), LAML (Acute Myeloid Leukemia) or LGG(Brain Lower Grade Glioma). Subsequently, the expression status of FAM107A across various cancer types of TCGA was analyzed using the Oncomine dataset. As shown in **Figure 1B**, FAM107A had a relatively lower expression in bladder cancer, brain and central nervous system (CNS) cancer, breast cancer, cervical cancer, colorectal cancer, esophageal cancer, gastric cancer, head and neck cancer, kidney cancer, lung cancer, melanoma, pancreatic cancer, prostate cancer, and sarcoma.

Using the “Pathological Stage Plot” module of GEPIA2, a correlation between FAM107A expression and the pathological stages of cancers was also detected, including KICH, KIRC, LIHC (Liver hepatocellular carcinoma), LUSC, SKCM (Skin Cutaneous Melanoma), STAD (all P<0.05) (**Figure 1C**). As for the expression of FAM107A protein, the CPTAC dataset was implemented to explore the difference between primary cancers and normal tissues. The results showed lower protein expression of FAM107A in lung adenocarcinoma and UCEC than in normal tissues (P<0.05) (**Figure 1D**).

To be comprehensive, we also combined the HPA, GTEx, and CCLE datasets to assess the expression level of FAM107A in nontumor tissues and various tumor cell lines. Highest expression of FAM107A mRNA was detected in the central nervous system for normal tissues, similar to the cerebral cortex, cerebellum, hippocampal amygdala, and basal ganglia. Meanwhile, a neglected expression of FAM107A was also observed in endocrine glands (thyroid gland and pituitary gland), lung and urogenital system (kidney, urinary bladder, epididymis, seminal vesicle, prostate, fallopian tube, endometrium, cervix, uterine and placenta) (**Figures S2A, B**). However, a quite low expression level was found in most tumor cell lines after analyzing the sequencing data from CCLE dataset (**Figure S2C**).

### Survival Analysis of FAM107A

**Figure 2:**
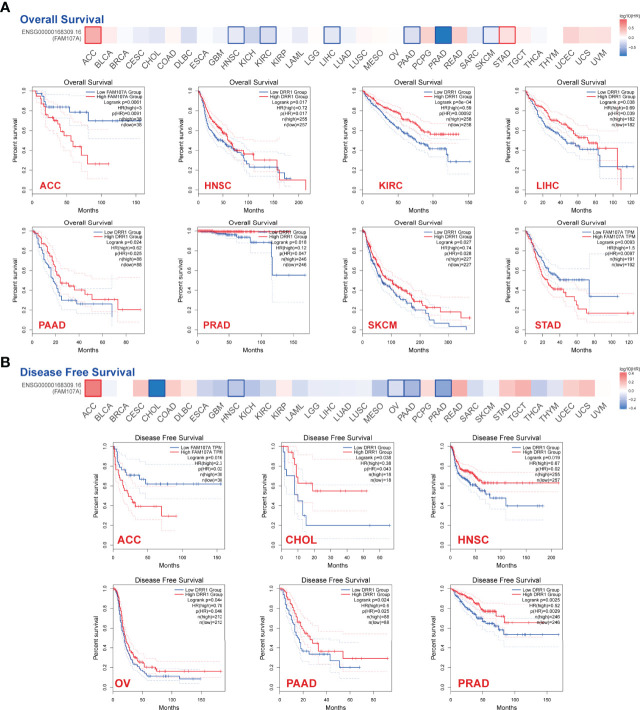
Correlation between expression level of FAM107A and survival prognosis of cancers in TCGA. Overall survival **(A)** and disease-free survival **(B)** analyses of FAM107A gene expression in different tumors from TCGA database were performed using GEPIA2 tool. The survival map and Kaplan-Meier curves with positive results are given.

To explore the prognostic value of FAM107A, the cancer cases were divided into high-expression and low-expression groups according to the median expression value of FAM107A for investigating the correlation of FAM107A expression with prognoses of patients across different tumor types by using the datasets from TCGA and GEPIA2. The results displayed that down-regulated FAM107A expression was associated with poor prognosis in many cancers. A correlation between low FAM107A expression and poor overall survival from the TCGA cases was detected in KIRC (HR=0.59, Logrank p<0.001), LIHC (HR=0.69, Logrank p=0.038), PAAD (Pancreatic adenocarcinoma) (HR=0.62, Logrank p=0.024), PRAD (HR=0.12, Logrank p=0.018), SKCM (HR=0.74, Logrank p=0.027), HNSC (HR=0.72, Logrank p=0.017) (**Figure 2A**). Besides, low FAM107A expression linked to poor prognosis as for disease-free survival (DFS) was discovered in CHOL (Cholangiocarcinoma) (HR=0.38, Logrank p=0.038), HNSC (HR=0.67, Logrank p=0.019), OV (HR=0.78, Logrank p=0.044), PAAD (HR=0.6, Logrank p=0.024), PRAD (HR=0.52, Logrank p=0.0025) (**Figure 2B**). Conversely, the high expression level of FAM107A was associated with poor OS for adrenocortical carcinoma (ACC) (HR=3, Logrank p=0.0061) and STAD (HR=1.5, Logrank p=0.0093), and high FAM107A expression was also related to poor DFS for ACC (HR=2.3, Logrank p=0.016) (**Figures 2A, B**). All these survival data presented a different association of FAM107A expression with the prognosis of different cancer cases.

### Genetic Alteration of FAM107A in Various Tumors

**Figure 3:**
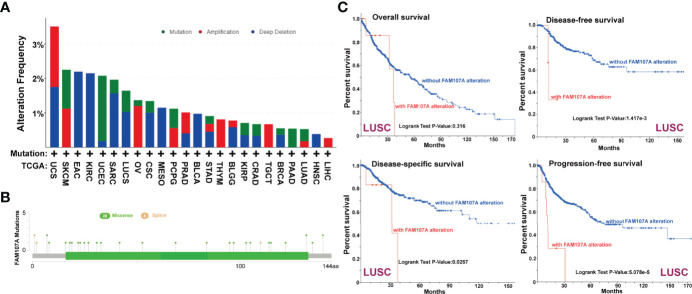
Genetic alteration of FAM107A in different tumors of TCGA. We analyzed the mutation features of FAM107A for the TCGA tumors using the cBioPortal tool. **(A)** The alteration frequency with mutation type of FAM107A was analyzed *via* cBioPortal tool. **(B)** The mutation sites of FAM107A gene were investigated using the cBioPortal tool. **(C)** The potential correlation between mutation status and overall, disease-specific, disease-free and progression-free survival of LUSC was analyzed with the cBioPortal tool.

**Figure 4:**
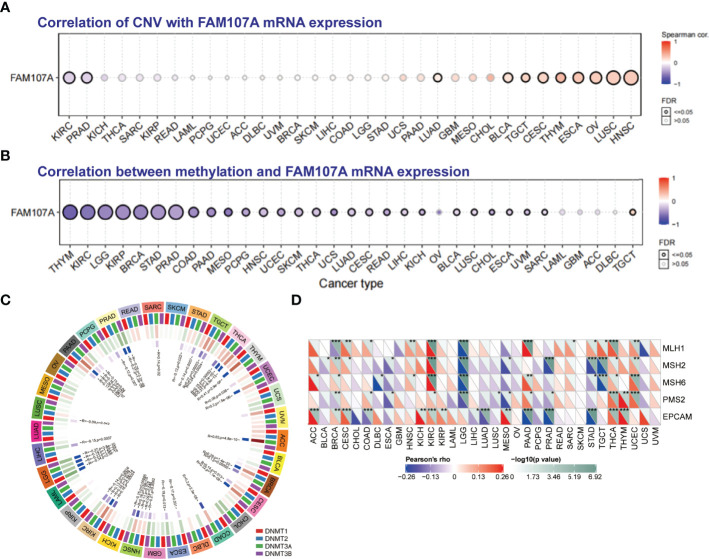
The correlation between FAM107A expression and CNV, DNA methylation and mutation levels of MMR genes. **(A)** The correlation between CNV and expression of FAM107A mRNA was analyzed. Blue dots indicate negative correlation and red dots indicate positive correlation between CNV and FAM107A expression. The darker color represents the higher correlation. The larger size of the point represents the greater significance. **(B)** The correlation between methylation level and expression of FAM107A mRNA was analyzed by the GSCA database. Blue dots indicate negative correlation and red dots indicate positive correlation between methylation and FAM107A expression. The darker color represents the higher correlation. The larger size of the point represents the greater significance. **(C)** The correlation between FAM107A expression and the expression levels of four methyltransferases was displayed. **(D)** The correlation between FAM107A mRNA expression and mutation levels of five key MMR genes (MLH1, MSH2, MSH6, PMS2, EPCAM) was analyzed based on TCGA data. The upper triangle in each tile indicates log10 transformed P-value, and the lower triangle indicates coefficients calculated by Pearson’s correlation test. *P < 0.05; **P < 0.01; ***P < 0.001.

To study the genetic alteration status of FAM107A in different tumors, the cBioPortal tool was applied to analyze FAM107A genetic alteration with data from TCGA dataset. As shown in **Figure S3**, four kinds of FAM107A genetic alteration were detected from all TCGA tumor samples with a total frequency of about 0.9%, including deep deletion, amplification, missense mutation, and splice mutation. Deep deletion was the main type of FAM107A genetic alteration. All FAM107A gene mutational sites and sample numbers are presented in **Figure 3A**. As for the specific tumors, uterine carcinosarcoma appeared the highest alteration frequency of FAM107A(>3%). Meanwhile, an alteration frequency exceeding 2% was determined in skin cutaneous melanoma, esophageal adenocarcinoma, kidney renal clear cell carcinoma, and uterine corpus endometrial carcinoma. The types, sites, and case numbers of the FAM107A genetic alteration are further presented in **Figure 3B**. Moreover, the potential correlation of FAM107A genetic alteration with the survival prognosis of different tumor cases was explored using the “Comparison” module of cBioPortal tool. The result showed a worse prognosis for LUSC patients with FAM107A genetic alteration with regard to disease-free survival(P = 1.417e-3), disease-specific survival(P = 0.0267) and progression-free survival(P = 5.078e-5), but not overall survival(P = 0.316) (**Figure 3C**). In addition, the correlation between FAM107A expression and tumor mutational burden (TMB)/microsatellite instability (MSI) was analyzed across all cancers of TCGA. The result showed a negative correlation between FAM107A expression and TMB existed in most cancers including BLCA (P = 0.018), BRCA (P = 0.02), HNSC (P = 0.044), KIRC (P= 0.0043), LGG (Brain Lower Grade Glioma) (P= 0.0013), LIHC (P=0.0028), LUAD (P= 1.5e-07), LUSC (P= 0.0028), PAAD (P= 6e-08), PRAD (P= 5.3e-27), READ (P= 0.0073), SKCM (P= 0.014), STAD (P= 5.7e-15), THCA (P= 0.007) and UCE C (P= 0.00021). However, a positive correlation between FAM107A expression and TMB was found in LAML (P = 0.048) and THYM (Thymoma) (P= 0.0062) (**Figure S5A**). As for MSI, we observed a negative correlation between FAM107A expression and MSI for CESC (P = 0.0058), ESCA (P = 0.0039), HNSC (P = 0.0012), LUSC (P= 0.043), OV (Ovarian serous cystadenocarcinoma) (P= 0.0041), PAAD (P=0.046), PRAD (P= 0.0014), SKCM (P= 0.0014), STAD(P= 9.8e-11) and UCS (P = 0.016) (**Figure S5B**). In-depth research should be carried out to determine this result. Interestingly, the expression of FAM107A was detected to have significant correlations with mutation levels of more than one key mismatch repair (MMR) genes (MLH1, MSH2, MSH6, PMS2, and EPCAM) in various cancers like BRCA, KIRC, and THCA (**Figure 4D**). Moreover, a statistically significant correlation between FAM107A CNV and its mRNA expression was observed in LUAD, BLCA, TGCT, CESC, THYM, ESCA, OV, LUSC and HNSC, which indicated CNV may be one of the important mechanisms leading to the alteration of FAM107A gene expression (**Figure 4A**).

### Epigenetic Alteration of FAM107A Gene

To identify whether DNA methylation plays an essential role in regulating the gene expression of FAM107A, we firstly applied the GSCALite database to analyze the correlation between DNA methylation of FAM107A and expression in various cancers. As shown in **Figure 4B**, the expression of FAM107A was found to be negatively correlated with DNA methylation in multiple cancers, mainly including THYM, KIRC, LGG, KIRP, BRCA, STAD, PRAD, COAD, PAAD, MESO(Mesothelioma), PCPG(Pheochromocytoma and Paraganglioma), HNSC, UCEC, SKCM, THCA and LUAD. Subsequently, using the DiseaseMeth(version 2.0) database, we investigated the differential DNA methylation level between tumors and normal tissues in pan-cancers. The results showed a higher DNA methylation level existed in many cancers than in corresponding normal tissues, including COAD, pilocytic astrocytoma tumor, READ, nasopharyngeal carcinoma, CHOL, LUAD, LUSC, LIHC, oral squamous cell carcinoma, KIRC, esophageal squamous cell carcinoma, ESCA, HNSC and PAAD(**Figure S4**). However, DNA methylation level was detected anomaly higher in normal tissues than LGG. Furthermore, we assessed the correlations between gene expression of four DNA-methyltransferases (DNMT1, DNMT2, DNMT3A, and DNMT3B) and expression of FAM107A mRNA. As shown in **Figure 4C**, in multiple kinds of cancers including ACC, BRCA, CESC, COAD, ESCA, HNSC, KIRC, LGG, LIHC, LUSC, PAAD, PRAD, SARC, STAD, TGCT, THCA, THYM and UCEC, FAM107A was observed to have an expressive correlation with at least one type of DNA-methyltransferases.

### Immune Infiltration Analysis of FAM107A

**Figure 5:**
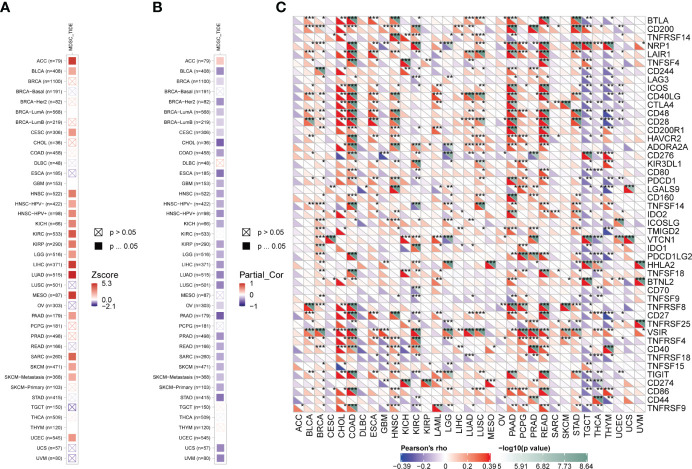
Immune infiltration analysis of FAM107A. **(A)** The correlation between the infiltration level of MDSCs and overall survival was explored across all types of cancer in TCGA. **(B)** The potential correlation between the expression level of the FAM107A gene and the infiltration level of MDSCs was explored across all types of cancer in TCGA. **(C)** The correlation between FAM107A mRNA expression and the expression levels of key immune checkpoints in different cancers from the TCGA database was investigated. *P < 0.05; **P < 0.01; ***P < 0.001.

Accumulating evidence indicates that tumor immune microenvironment plays an important role in the occurrence, progression, and metastasis of tumors. Therefore, we used the SangerBox tool to investigate the correlation of FAM107A expression with Stromal, Immune, and ESTIMATE score in pan-cancers, which represented the abundance of stromal components, immune components, and tumor purity to some extent. According to rank of the relationship between the expression level and the score, scatter plots of top nine tumors of Stromal, Immune, and ESTIMATE score were displayed, respectively (**Figures S6**–**S8**). Then, we applied the TIMER2.0 database to explore the potential relationship between the FAM107A expression and infiltration level of diverse immune cells in multiple cancers of TCGA. It is worth noting that the immune infiltration of myeloid derived suppressor cells(MDSCs) was negatively correlated with prognosis in many cancers (**Figure 5A**). Herein, we investigated the correlation between the FAM107A expression and infiltration level of MDSCs, and the results showed a statistically negative correlation between them in many tumors as expected (**Figure 5B**). Moreover, we further assessed the expression correlations between FAM107A and immune checkpoints in pan-cancers. As shown in **Figure 5C**, the FAM107A expression was robustly and significantly correlated with recognized immune checkpoints in most cancers including BLCA, BRCA, CHOL, COAD, HNSC, KIRC, PAAD, READ, STAD, and TGCT.

### Enrichment Analysis of FAM107A

**Figure 6:**
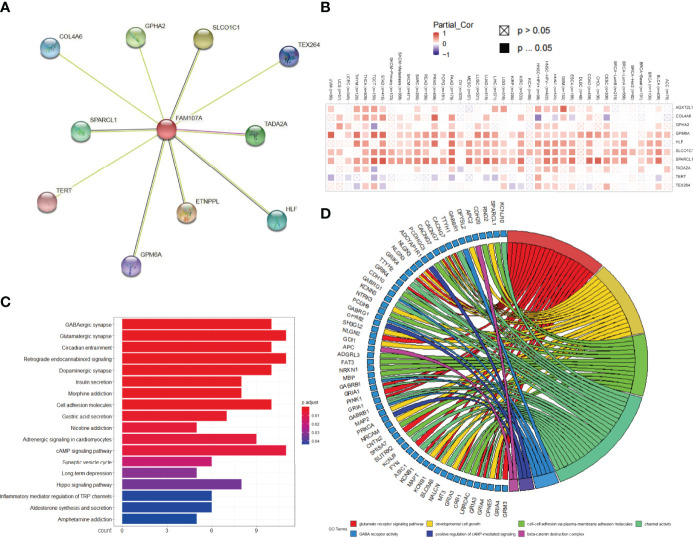
Analysis of FAM107A-related gene enrichment. **(A)** Available determined FAM107A-binding proteins was obtained with the STRING tool. **(B)** The heatmap of expression correlation between FAM107A and its binding proteins in the different cancer types are displayed. **(C)** KEGG pathway analysis was performed based on the FAM107A-binding and correlated genes. **(D)** The chordal graph for the potential cancer-related terms in GO analysis is shown.

To explore the potential molecular function of FAM107A involved in occurrence, progression, or metastasis of tumors, we combined String and GEPIA2 tools to obtain the FAM107A-binding proteins and expression-correlated genes for enrichment analyses and 10 FAM107A-binding proteins were proved by experimental evidence *via* STRING tool (**Figure 6A**). Using the TIMER2.0 database, we performed a heatmap to show the expressive correlation between FAM107A and these 10 corresponding genes in diverse cancers. As shown in **Figure 6B**, the expression of FAM107A was detected in most cancers to be significantly positively correlated with four of above genes including GPM6A(Glycoprotein M6A), HLF(Hepatic leukemia factor), SLCO1C1(Solute carrier organic anion transporter family, member 1C1), SPARCL1(Secreted protein acidic and rich in cysteines-like protein 1). Afterwards, we used the GEPIA2 tool to obtain top 300 FAM107A-correlated genes by combining all tumor expression data of TCGA. Subsequently, KEGG and GO enrichment analyses were conducted with the union of the 10 FAM107A-targeting genes and 300 expression-correlated genes. The KEGG data indicated that many genes are linked to the pathways of “Cell adhesion molecules” and “cAMP signaling pathway” (**Figure 6C**). Likewise, the result showed that “developmental cell growth”, “cell-cell adhesion *via* plasma-membrane adhesion” and “positive regulation of cAMP mediated signaling” were enriched in GO terms and might be in involved in the impact of FAM107A on tumor pathogenesis (**Figure 6D**).

### Knockdown of FAM107A Promoted the Malignant Behaviors in Bladder Cancer and Renal Cancer Cells

**Figure 7:**
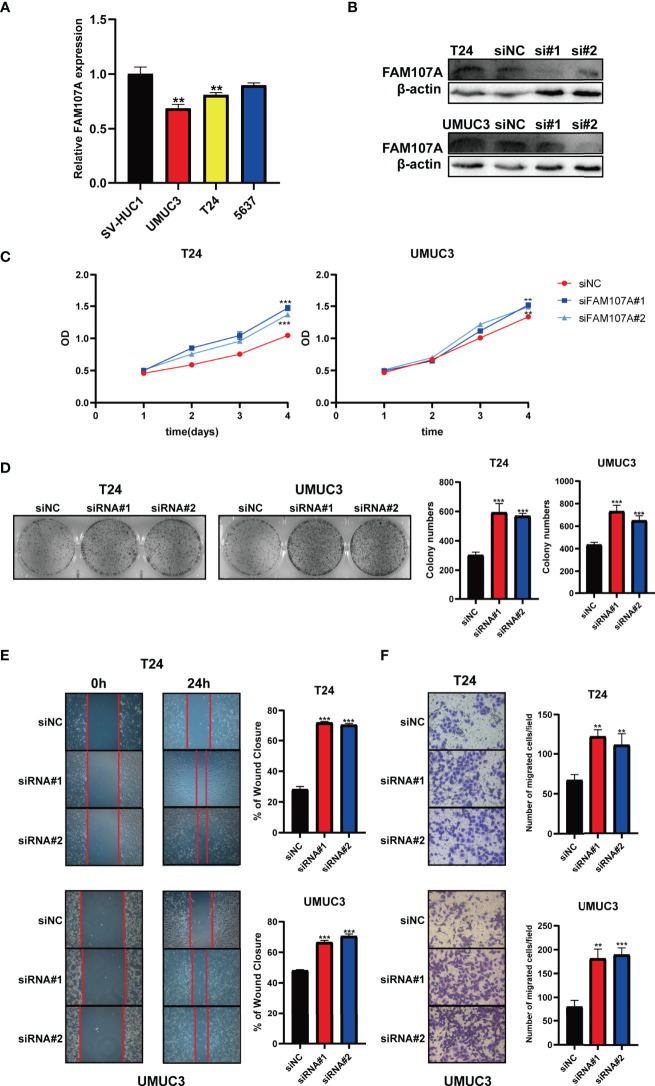
Knockdown of FAM107A promoted the proliferation, migration, and invasion of bladder cancer cells. **(A)** The expression level of FAM107A mRNA was evaluated in SV-HUC1 and various bladder cancer cell lines by qRT-PCR. **(B)** T24 and UM-UC3 cells were transfected with si-FAM107A, the knockdown was validated by Western blot. The proliferation of bladder cancer cells was examined by CCK-8 assay **(C)** and colony-formation assay **(D)**. **(E)** The migration of bladder cancer cells was examined by wound healing assay. **(F)** The invasion of bladder cancer cells was explored by transwell assay. Data are shown as mean ± SD. **P < 0.01; ***P < 0.001.

To further investigate the effect of FAM107A on bladder cancer, we firstly examined the expression of FAM107A in multiple bladder cancer cell lines and renal cancer cell lines. As shown in **Figure 7A**, a relatively lower expression of FAM107A was detected in UMUC3 and T24 cells compared with SV-HUC cells. Likewise, a lower expression of FAM107A was also noted in renal cancer cell lines (**Figure S9A**). Subsequently, we downregulated the FAM107A expression in UMUC3, T24, 786-O and 769-P cells *via* two FAM107A siRNAs. The successful silence of FAM107A expression in these four cell lines was validated at a translational level (**Figure 7B**, **Figure S9B**). Next, CCK8 assay was carried out to evaluate cell proliferation. The results suggest a significant promotion of proliferation rates in the tumor cells with FAM107A downregulation (**Figure 7C** and **Figure S9C**). Colony-formation assay also confirmed the positive effect of siFAM107A on bladder cancer cell proliferation (**Figure 7D**, **Figure S9D**). Finally, wound healing and Transwell assays were performed to investigate the effect of FAM107A on the motility of bladder cancer cells. There was a significant reduction in both migration and invasion in tumor cells after knockdown of FAM107A (**Figures 7E, F**, **Figures S9E, F**). In summary, downregulated FAM107A facilitates the proliferation, migration, and invasion in bladder cancer and renal cancer cells.

## Discussion

Accumulating evidence indicates that FAM107A may be an important biological protein in organisms. To date, FAM107A has been verified to take part in the normal function of the nervous system and the development of tumors. Binding with F-actin and participating in the regulation of numerous physiological processes is the widely recognized mechanism of FAM107A (26–28). Although the expression of FAM107A mRNA was observed downregulation in multiple cancers, studies show dual effects of “tumor suppression” and “tumor promotion” of FAM107A in the process of tumor genesis and development. In glioblastoma, FAM107A was downregulated during tumorigenesis and upregulated in the process of tumor invasion, which promotes cell invasion and epithelial-mesenchymal transformation through the activation of AKT (27). Nevertheless, FAM107A attached to F-actin and formed a stable complex with copper metabolism MURR1 domain-containing 1(COMMD1), which accelerated the ubiquitination and degradation of NF-κB by promoting the polyubiquitination of a subunit of NF-κB. The degradation of NF-κB prevented the transition from G1 phase to S phase of tumor cell and thereby inhibited cell proliferation (12). The dual effects of FAM107A in malignant tumors are comparable to that of transforming growth factor-β(TGF-β) (29–31). In addition, the expression of FAM107A was influenced by some non-coding RNAs in cancer. Di Wang et al. demonstrated microRNA-146b-3p could facilitate the progression and metastasis of colorectal cancer *via* interfering with the expression of FAM107A (32). Nevertheless, we failed to find any comprehensive cancer-related study of FAM107A through a literature search. Since FAM107A was closely related to multiple cancers and acted as an important potential effector, a pan-cancer analysis of FAM107A was performed and the effect of FAM107A in bladder cancer cells was explored for the first time in this study.

In the present study, the expression level of FAM107A in normal tissues and tumor cell lines was analyzed *via* HPA, GTEx, and CCLE datasets. We observed that FAM107A was predominantly expressed in the central nervous system and fairly expressed in endocrine glands, lung, and urogenital system (**Figures S2A, B**). On the contrary, FAM107A demonstrated low expression in most tumor cell lines (**Figure S2C**). Then, we evaluated the differential expression levels of FAM107A mRNA between tumor and normal tissues in the Oncomine and TCGA databases. The expression of FAM107A gene was reduced in most tumors (**Figures 1A, B**). In addition, the expression of the FAM107A protein was also observed to be downregulated in lung adenocarcinoma and UCEC (**Figure 1D**). However, an upregulation of FAM107A was discovered in THYM and lymphoma (**Figure 1B**, **Figure S1**). Although we found a significantly negative correlation between FAM107A expression and promoter methylation in THYM, we did not discover an upregulating promoter methylation in THYM (**Figure 4B**). Besides, a combined linkage and association analysis of classical Hodgkin lymphoma showed low or absent FAM107A expression in Hodgkin Reed-Sternberg cell lines and in Hodgkin Reed-Sternberg cells in Hodgkin lymphoma tissue (19). We suspect the expression of FAM107A was compensatory increase in these tumors because of its incomplete inactivation. Next, we applied GEPIA2 tool to explore the prognostic value of FAM107A of TCGA datasets. A statistical correlation between low expression of FAM107A and poor overall survival prognosis was detected in KIRC(HR=0.59, Logrank p<0.001), LIHC(HR=0.69, Logrank p=0.038), PAAD(HR=0.62, Logrank p=0.024), PRAD(HR=0.12, Logrank p=0.018), SKCM(HR=0.74, Logrank p=0.027), HNSC(HR=0.72, Logrank p=0.017) (**Figure 2**). All results above suggest that FAM107A might serve as a novel anti-oncogene in these tumor types.

We then used the cBioPortal tool to study the genetic alteration of FAM107A in cancers. Our analysis showed a low mutation frequency (0.9%) of FAM107A in tumor cases from TCGA with deep deletion as the main type of FAM107A genetic alteration (**Figure 3A**, **Figure S3**). Moreover, we found a correlation between FAM107A genetic alteration and worse prognosis for LUSC patients with regard to disease-free survival (P = 1.417e-3), disease-specific survival(P = 0.0267) and progression-free survival(P = 5.078e-5), but not overall survival(P = 0.316) (**Figure 3C**). The data indicates that genetic alteration was not the main reason for FAM107A dysregulated expression. As gene methylation is a well-known factor which influences the gene expression, we continued to analyze the DNA methylation of FAM107A gene *via* GSCALite database. The result showed a negative correlation between FAM107A expression and promoter methylation in various cancers (**Figure 4B**). Furthermore, a higher DNA methylation level of FAM107A was demonstrated in numerous cancers compared with adjacent tissues (**Figure S4**). Notably, the expression of FAM107A was significantly correlated with the expression of DNA-methyltransferases in multiple cancers (**Figure 4C**). These implied that the dysregulated expression of FAM107A was mainly influenced by promoter methylation in cancers. The MMR system plays important roles in maintaining the stability and integrity of genome (33, 34). Additionally, TMB and MSI are served as novel sensitive predictors of immunotherapy with a profound impact on tumor phenotype and patient survival (35, 36). In the current study, the potential association between the expression of FAM107A and MMR, MSI or TMB was analyzed. The results revealed that the expression of FAM107A was enormously related to MMR genes expression in various tumors (**Figure 4D**). Further analysis showed that the expression of FAM107A was significantly negatively correlated with TMB in 15 cancer types and MSI in 10 cancer types (**Figure S5**). The results indicated that FAM107A might be a vital factor which could influence or predict the response of cancer patients to immunotherapy.

Given that tumor-infiltrating immune cells participate in the initiation and progression of cancer, we agreed to explore the potential relationship between FAM107A gene expression and the infiltration level of different immune cells in diverse cancers of TCGA. As a result, a statistically negative correlation was observed in various cancers between FAM107A gene expression and the infiltration level of MDSCs, which was supposed to be a risk factor to the prognosis of cancer patients according to the results of survival analysis (**Figures 5A, B**). MDSCs, a group of heterogeneous cells derived from bone marrow, which are the precursors of dendritic cells (DCs), macrophages and granulocytes, have the ability to significantly inhibit the immune cell response (37). Besides, MDSCs were observed to produce VEGF, bFGF, Bv8, and MMP9 to promote tumor progression by influencing the tumor microenvironment and tumor angiogenesis (38–40). Therefore, FAM107A may inhibit tumor progression by regulating the activity of MDSCs. Moreover, correlation analysis showed the expression of FAM107A was correlated with immune checkpoints in diverse cancers with statistical significance (**Figure 5C**). Therefore, FAM107A may be associated with the regulation of the tumor microenvironment. Further studies should be undertaken to investigate the explicit mechanisms. Integrating information on FAM107A-binding proteins and FAM107A expression-related genes across all tumors, GO and KEGG enrichment analyses were conducted to investigate the potential functional role of FAM107A in cancers. Interestingly, we identified “Cell adhesion” and “cAMP signaling pathway” terms as the potential impact of FAM107A on the etiology or pathogenesis of cancers both in GO and KEGG analyses (**Figure 6**). Also, FAM107A may affect the progression of cancer through AKT or NF-κB pathway (12, 27, 28).

Furthermore, a series of experiments *in vitro* were conducted to investigate the impact of FAM107A on bladder cancer and renal cancer. The analyses demonstrated a relatively lower expression of FAM107A in bladder cancer and renal cancer cell lines. In addition, FAM107A knockdown promoted the proliferation, migration, and invasion of the cancer cell lines (**Figure 7**, **Figure S9**). That said, FAM107A may be served as an anti-cancer gene in bladder cancer and renal cancer.

There were certain limitations in this study. Most bioinformatics analyses were conducted based on the expression of FAM107A at the mRNA level, so the conclusions were deduced lacking ample supporting data FAM107A translational level. Conversely, although we confirmed the biological effect of FAM107A on bladder cancer cells, the explicit regulatory mechanisms remain unclear. Therefore, further studies should be conducted to explore the exact signal pathway regulated by FAM107A and validate our conclusions.

Taken together, our pan-cancer analyses of FAM107A confirmed the expression pattern and prognostic value across multiple cancers. In addition, we found statistical correlations of FAM107A expression with DNA methylation, immune cell infiltration, immune checkpoints, and tumor purity. Importantly, FAM107A was recognized as a novel potential tumor-suppressor gene in bladder cancer and renal cancer by inhibiting the progression of cancer.

## Data Availability Statement

The original contributions presented in the study are included in the article/**Supplementary Material**. Further inquiries can be directed to the corresponding author.

## Author Contributions

DO, ZZ, and M-eL had the idea for this study. ZW supervised the acquisition of the data and helped DO with experiment *in vitro*. PS undertook the statistical analysis. DO wrote the first draft of the manuscript. YH, SLS and SFS commented on and critically revised the manuscript. All authors approved the final version of the manuscript.

## Funding

This work was supported by the Guangdong Medical Research Foundation (A2020223 and A2021435) and Supporting Program of the First Affiliated Hospital of Shantou University Medical College (202003-3).

## Conflict of Interest

The authors declare that the research was conducted in the absence of any commercial or financial relationships that could be construed as a potential conflict of interest.

## Publisher’s Note

All claims expressed in this article are solely those of the authors and do not necessarily represent those of their affiliated organizations, or those of the publisher, the editors and the reviewers. Any product that may be evaluated in this article, or claim that may be made by its manufacturer, is not guaranteed or endorsed by the publisher.

## Supplementary Material

Supplementary Figure 1 Expression level of FAM107A gene in the tumors in the TCGA project and the corresponding normal tissues of the GTEx database.

Supplementary Figure 2 MRNA expression levels of FAM107A in normal tissues and tumor cell lines. MRNA expression levels of FAM107A in normal tissues in the HPA (Human protein atlas) dataset **(A)** and GTEx dataset **(B)**. **(C)** MRNA expression levels of FAM107A in various tumor cell lines from the Cancer Cell Line Encyclopedia(CCLE) database.

Supplementary Figure 3 The mutation spectrum of FAM107A across TCGA pan-cancer studies using cBioPortal Oncoprint. Each vertical bar represents a patient.

Supplementary Figure 4 Differential methylation level of FAM107A gene between tumor and corresponding normal tissues in 15 cancer types from DiseaseMeth database.

Supplementary Figure 5 Correlation between mRNA expression levels of FAM107A and tumor mutational burden (TMB) **(A)**, Microsatellite instability (MSI) **(B)** in various cancers from TCGA database.

Supplementary Figure 6 Correlation between mRNA expression levels of FAM107A and Stromal score in various cancers from TCGA database.

Supplementary Figure 7 Correlation between mRNA expression levels of FAM107A and Immune score in various cancers from TCGA database.

Supplementary Figure 8 Correlation between mRNA expression levels of FAM107A and ESTIMATE score in various cancers from TCGA database.

Supplementary Figure 9 Knockdown of FAM107A promoted the proliferation, migration and invasion of renal cancer cells. **(A)** The expression level of FAM107A mRNA was evaluated in 293T and various renal cancer cell lines by qRT-PCR. **(B)** 786-O and 769-P cells were transfected with si-FAM107A, the knockdown was validated by Western blot. The proliferation of renal cancer cells was examined by CCK-8 assay **(C)** and colony-formation assay **(D)**. **(E)** The migration of renal cancer cells was examined by wound healing assay. **(F)** The invasion of renal cancer cells was explored by transwell assay. Data are shown as mean ± SD. *P<0.05; **P < 0.01; ***p < 0.001.

## Glossary

**Table:**

ACC	Adrenocortical carcinoma
BLCA	Bladder Urothelial Carcinoma
BRCA	Breast invasive carcinoma
CESC	Cervical squamous cell carcinoma and endocervical adenocarcinoma
CHOL	Cholangiocarcinoma
COAD	Colon adenocarcinoma
CCLE	Cancer Cell Line Encyclopedia
CNA	Copy number alteration
CNV	Copy number variation
CPTAC	Clinical Proteomic Tumor Analysis Consortium
DLBC	Lymphoid Neoplasm Diffuse Large B-cell Lymphoma
DFS	Disease-free survival
DSS	Disease-specific survival
DRR1	Down-regulated in renal cell carcinoma 1
ESCA	Esophageal carcinoma
FAM107A	Family with sequence similarity 107, member A
GBM	Glioblastoma multiforme
GTEx	Genotype-tissue expression
GEO	Gene Expression Omnibus
GO	Gene ontology
GEPIA2	Gene expression profiling interactive analysis, version 2
HNSC	Head and Neck squamous cell carcinoma
HRP	Horseradish Peroxidase
IARC	International Agency for Research on Cancer
KICH	Kidney Chromophobe
KIRC	Kidney renal clear cell carcinoma
KIRP	Kidney renal papillary cell carcinoma
KEGG	Kyoto encyclopedia of genes and genomes
LGG	Brain Lower Grade Glioma
LIHC	Liver hepatocellular carcinoma
LUAD	Lung adenocarcinoma
LUSC	Lung squamous cell carcinoma
MDSC	myeloid-derived suppressor cell
MESO	Mesothelioma
MMR	mismatch repair gene
MSI	microsatellite instability
OV	Ovarian serous cystadenocarcinoma
OS	Overall survival
PAAD	Pancreatic adenocarcinoma
PCPG	Pheochromocytoma and Paraganglioma
PFS	Progression-free survival
PRAD	Prostate adenocarcinoma
READ	Rectum adenocarcinoma
SARC	Sarcoma
RIPA	Radio Immunoprecipitation Assay
SKCM	Skin Cutaneous Melanoma
STAD	Stomach adenocarcinoma
TBST	Tris Buffered Saline with Tween
TU3A	Tohoku University cDNA clone A on chromosome 3
TIMER2.0	Tumor immune estimation resource, version 2
TCGA	The Cancer Genome Atlas
TGCT	Testicular Germ Cell Tumors
THCA	Thyroid carcinoma
TMB	Tumor mutational burden
TIDE	Tumor Immune Dysfunction and Exclusion
THYM	Thymoma
UCEC	Uterine Corpus Endometrial Carcinoma
UCS	Uterine Carcinosarcoma
