# Supplementary figures and images for: Identification of the Putative Tumor Suppressor Characteristics of FAM107A via Pan-Cancer Analysis
Source: Front Oncol. 2022 May 20;12:861281. doi: 10.3389/fonc.2022.861281 (PMC9163664; doi:10.3389/fonc.2022.861281)

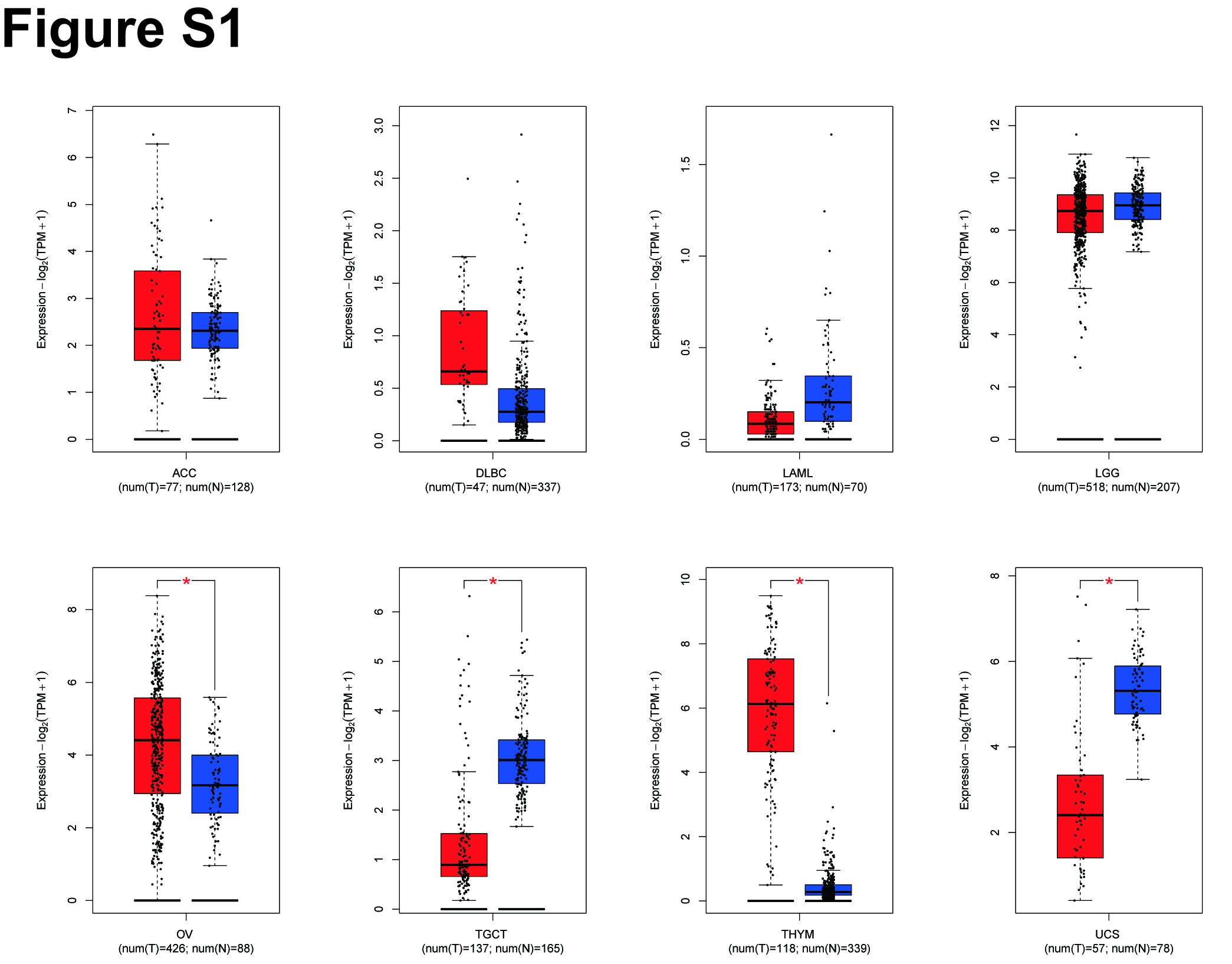

Supplement: Supplementary Figure 1 — Expression level of FAM107A gene in the tumors in the TCGA project and the corresponding normal tissues of the GTEx database. [file Image_1.tif]

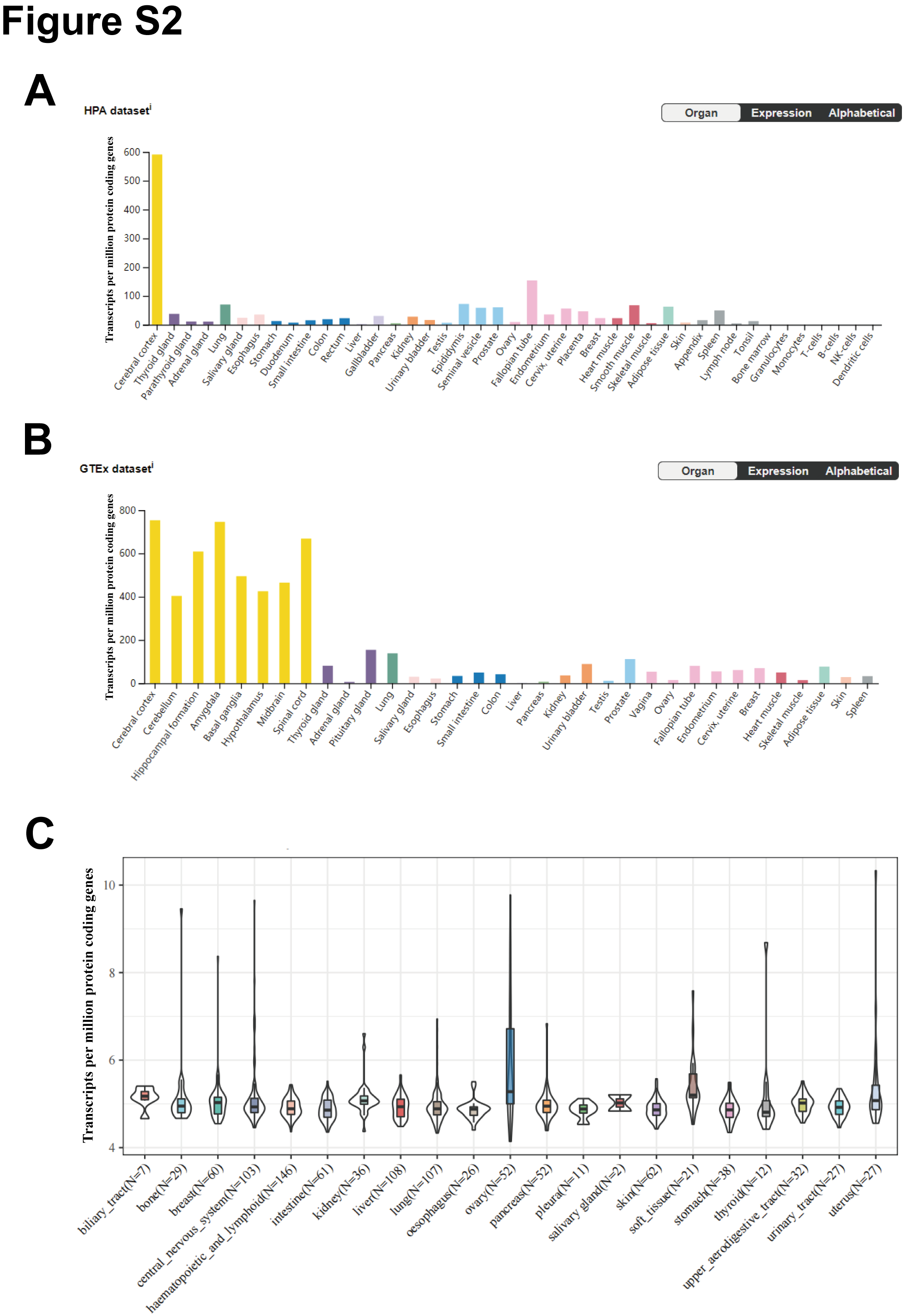

Supplement: Supplementary Figure 2 — MRNA expression levels of FAM107A in normal tissues and tumor cell lines. MRNA expression levels of FAM107A in normal tissues in the HPA (Human protein atlas) dataset (A) and GTEx dataset (B). (C) MRNA expression levels of FAM107A in various tumor cell lines from the Cancer Cell Line Encyclopedia(CCLE) database. [file Image_2.tif]

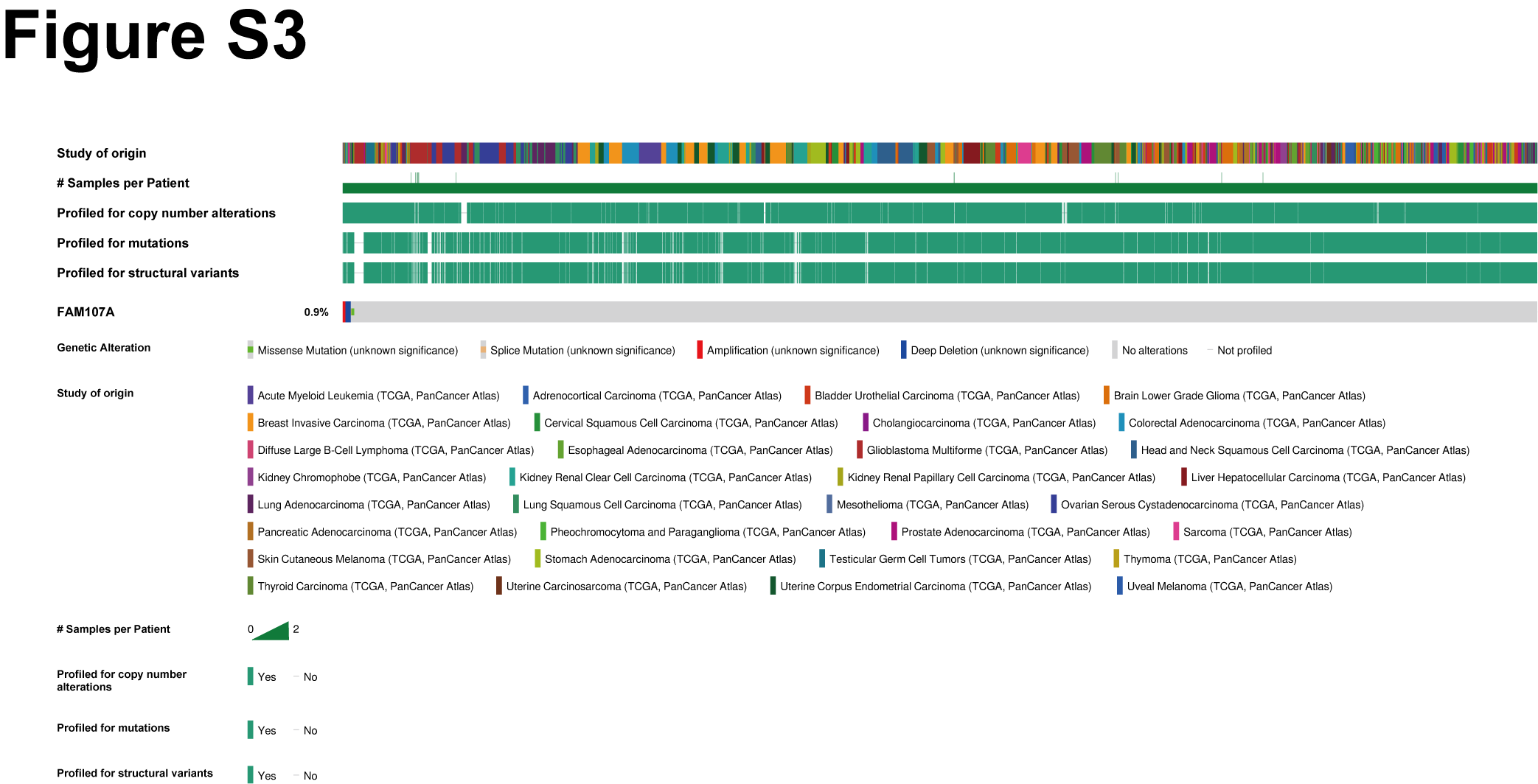

Supplement: Supplementary Figure 3 — The mutation spectrum of FAM107A across TCGA pan-cancer studies using cBioPortal Oncoprint. Each vertical bar represents a patient. [file Image_3.tif]

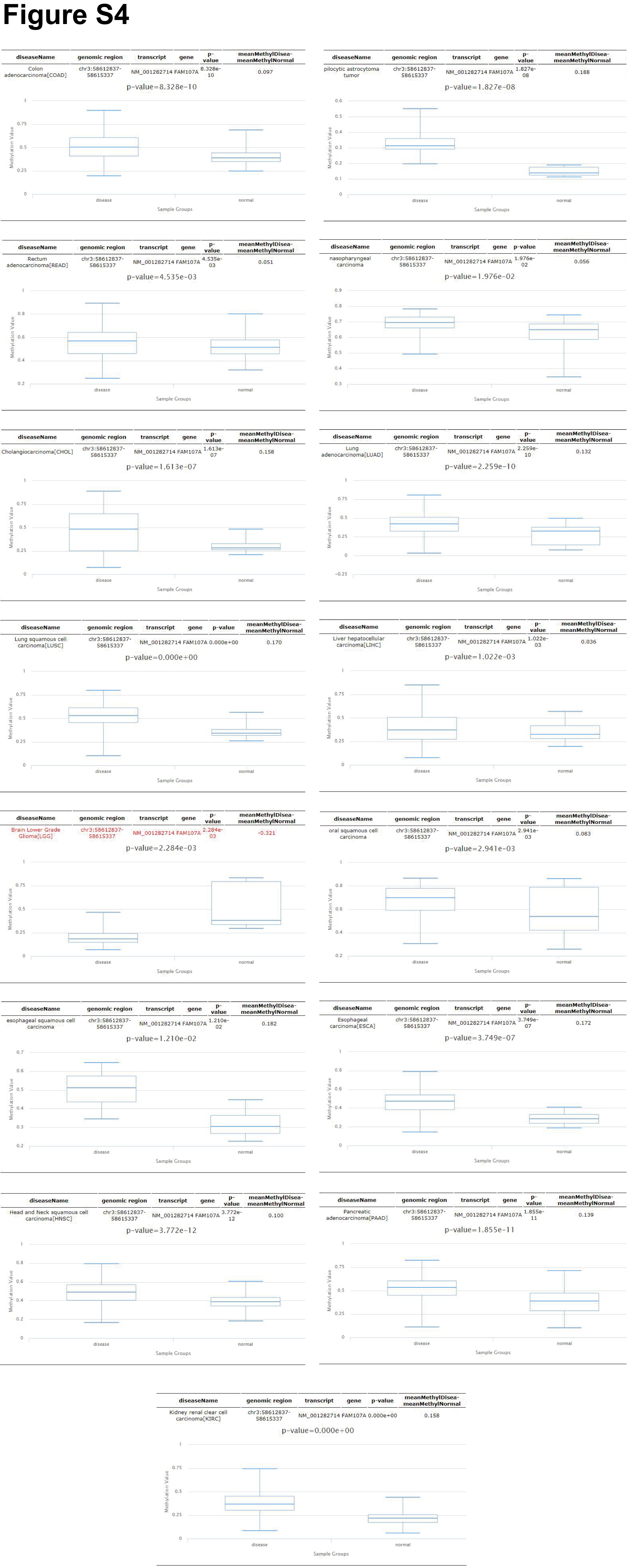

Supplement: Supplementary Figure 4 — Differential methylation level of FAM107A gene between tumor and corresponding normal tissues in 15 cancer types from DiseaseMeth database. [file Image_4.tif]

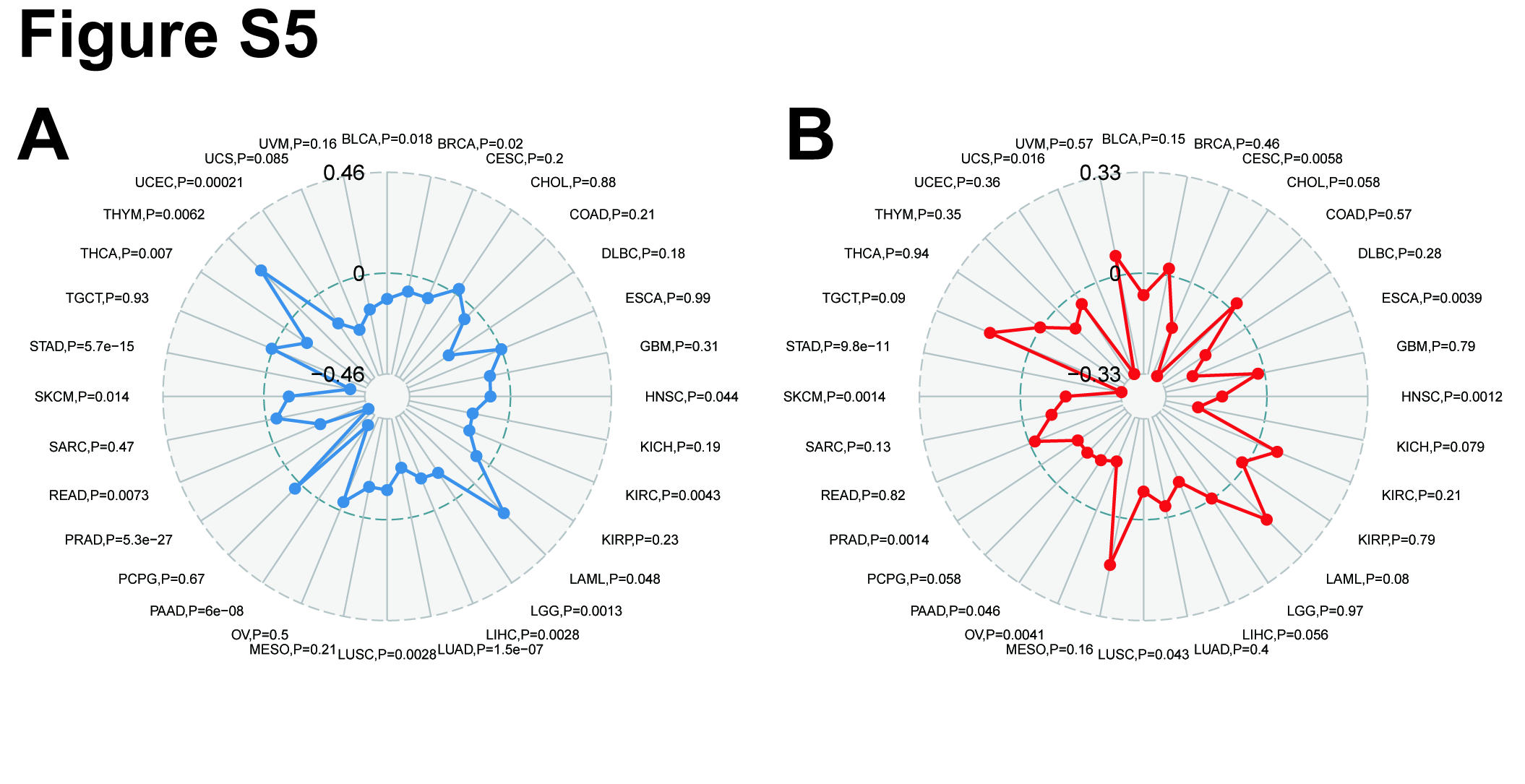

Supplement: Supplementary Figure 5 — Correlation between mRNA expression levels of FAM107A and tumor mutational burden (TMB) (A), Microsatellite instability (MSI) (B) in various cancers from TCGA database. [file Image_5.tif]

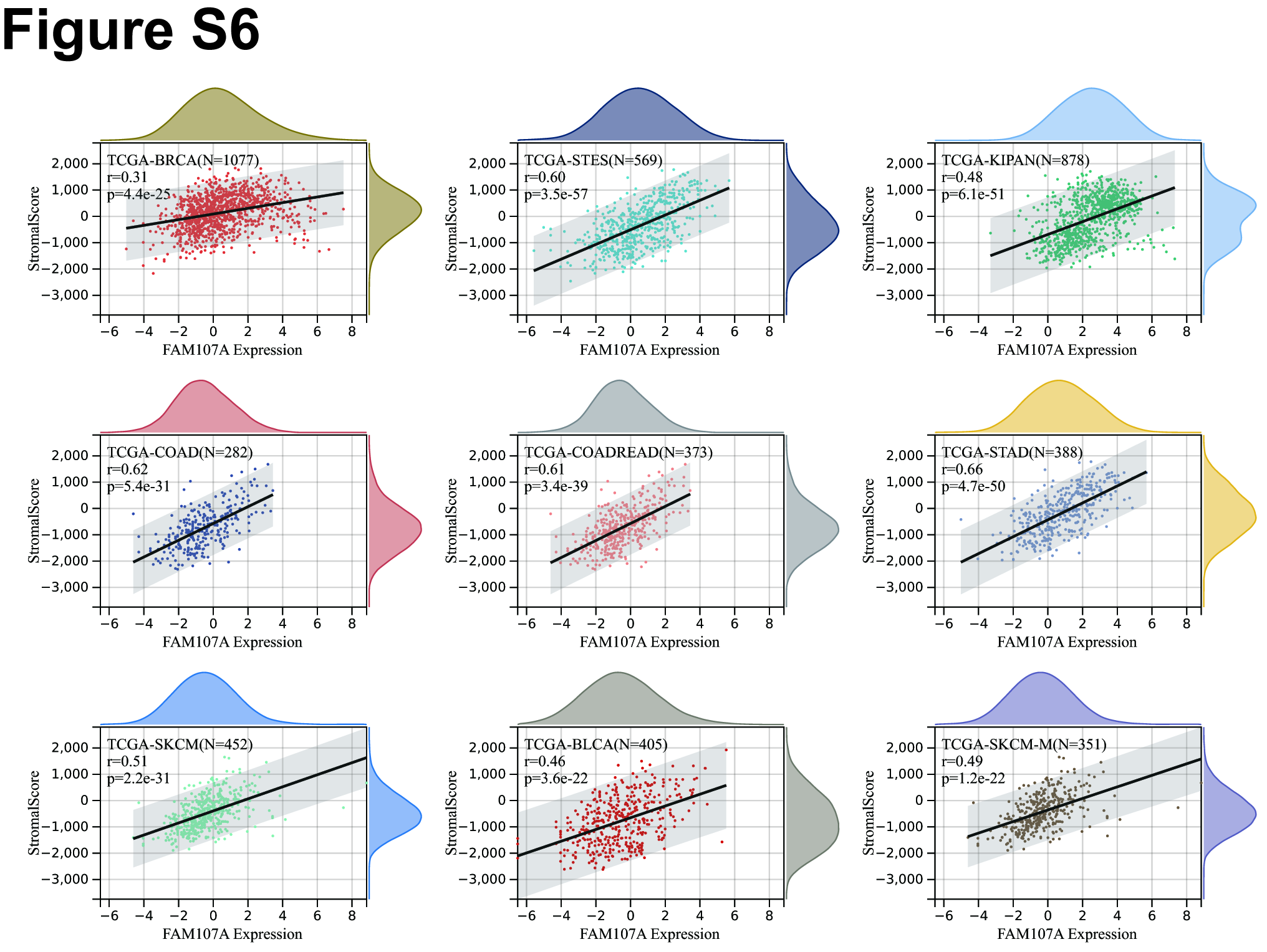

Supplement: Supplementary Figure 6 — Correlation between mRNA expression levels of FAM107A and Stromal score in various cancers from TCGA database. [file Image_6.tif]

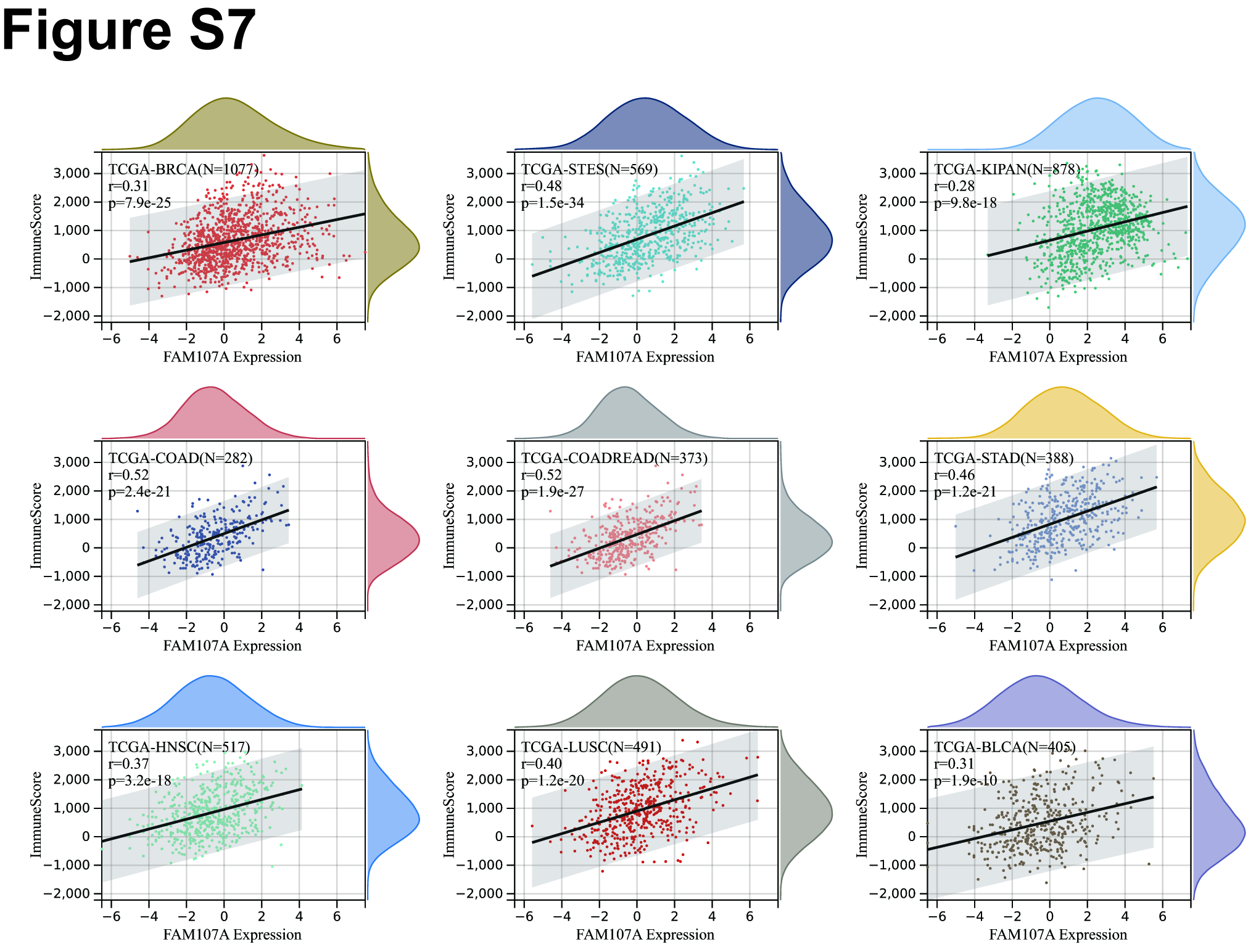

Supplement: Supplementary Figure 7 — Correlation between mRNA expression levels of FAM107A and Immune score in various cancers from TCGA database. [file Image_7.tif]

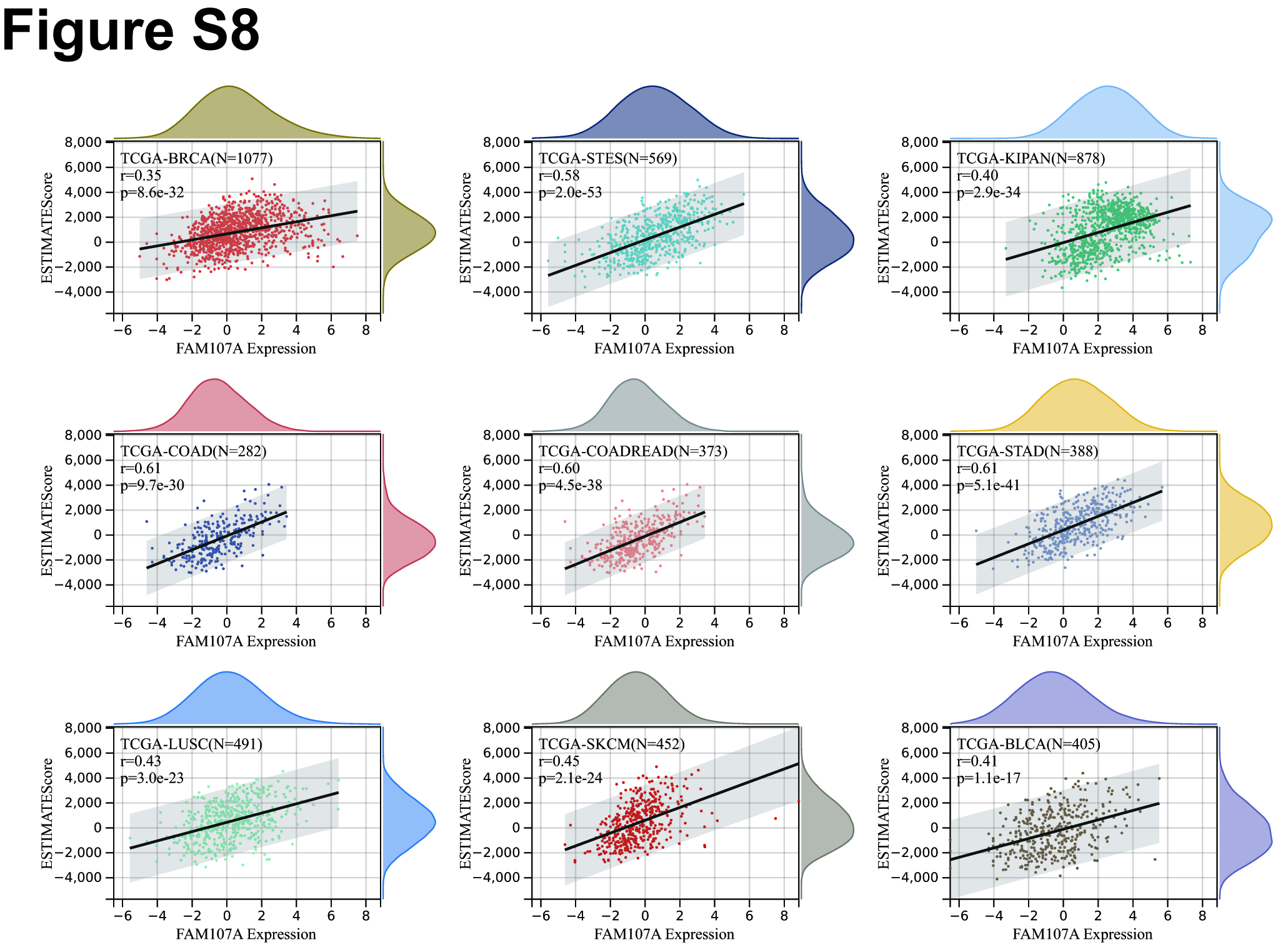

Supplement: Supplementary Figure 8 — Correlation between mRNA expression levels of FAM107A and ESTIMATE score in various cancers from TCGA database. [file Image_8.tif]

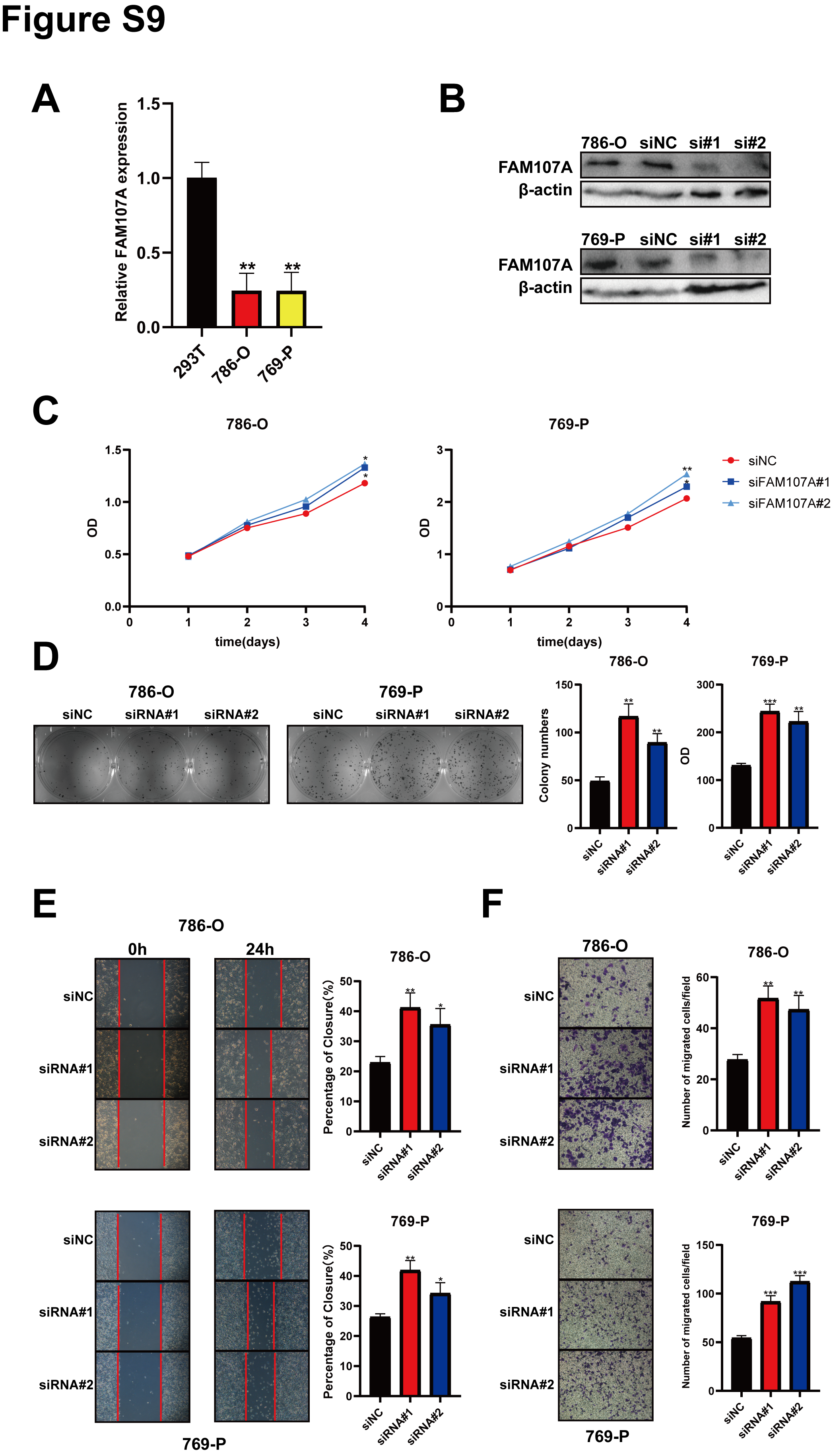

Supplement: Supplementary Figure 9 — Knockdown of FAM107A promoted the proliferation, migration and invasion of renal cancer cells. (A) The expression level of FAM107A mRNA was evaluated in 293T and various renal cancer cell lines by qRT-PCR. (B) 786-O and 769-P cells were transfected with si-FAM107A, the knockdown was validated by Western blot. The proliferation of renal cancer cells was examined by CCK-8 assay (C) and colony-formation assay (D). (E) The migration of renal cancer cells was examined by wound healing assay. (F) The invasion of renal cancer cells was explored by transwell assay. Data are shown as mean ± SD. *P<0.05; **P < 0.01; ***p < 0.001. [file Image_9.tif]
